# Supplementary material for: Rapid turnover of life-cycle-related genes in the brown algae
Source: Genome Biol. 2019 Feb 14;20:35. doi: 10.1186/s13059-019-1630-6 (PMC6374913; doi:10.1186/s13059-019-1630-6)
Supplement: Supplementary file 3 — Protein alignments, corresponding Gblocks html file and CDS sequences for the lineage conserved gametophyte-biased genes. (ZIP 2109 kb) [file 13059_2019_1630_MOESM3_ESM.zip › Con_GA_alignments/Ec-10_002060.aa_ali.fasta-gb.htm]

Ec-10\_002060.aa.fasta.trans.aa\_ali.fasta


## Gblocks 0.91b Results

Processed file: **Ec-10\_002060.aa.fasta.trans.aa\_ali.fasta**  
Number of sequences: **2**  
Alignment assumed to be: **Protein**  
New number of positions: **470** (selected positions are underlined in blue)

```
                         10        20        30        40        50        60
                 =========+=========+=========+=========+=========+=========+
Ec-10_002060     MRPGDGVRGVARPGRGDDVDLEALQDAFLRSAERPSAKVSRAKPPTIKAGSSLGVRKEDD
MSTRG.30551.1    ------------------------------------------------------------
                                                                             


                         70        80        90       100       110       120
                 =========+=========+=========+=========+=========+=========+
Ec-10_002060     DPRSGRGGADTANAPRSTGGGAAAATAVAAATAISPGTQPTRDNNNREPPRSLGVPPEER
MSTRG.30551.1    ------------------------------------------------------------
                                                                             


                        130       140       150       160       170       180
                 =========+=========+=========+=========+=========+=========+
Ec-10_002060     EKGPLTSASSPTGGMILNAPGVVSKSVREREVKPPAPMRIGGFAGGAGAGGAIGRRGDKQ
MSTRG.30551.1    ------------------------------------------------------------
                                                                             


                        190       200       210       220       230       240
                 =========+=========+=========+=========+=========+=========+
Ec-10_002060     RFGLAKGAKHVTAVAERGIIGGFPAVRHRSEMGLVKLGVGAAAASTGAPPATAAAAAAAA
MSTRG.30551.1    ------------------------------------------------------------
                                                                             


                        250       260       270       280       290       300
                 =========+=========+=========+=========+=========+=========+
Ec-10_002060     KGNAATERGGRGGAGRGGGGGVGNIPGDIHEENLSRISAMSPDEIADAQQEIRAALPPGA
MSTRG.30551.1    ------------------------------------------------------------
                                                                             


                        310       320       330       340       350       360
                 =========+=========+=========+=========+=========+=========+
Ec-10_002060     LEMLLRRGRGGGGKTVSGTPAPSAAAVAAAAAAAAAAAAAAAAAATAGETKAGDEENHCA
MSTRG.30551.1    ------------------------------------------------------------
                                                                             


                        370       380       390       400       410       420
                 =========+=========+=========+=========+=========+=========+
Ec-10_002060     GRRRSPVPATTGHGESAAPAGDEGDSSNGGGPSRSTATASATGEAAAAAAAAAAAAGDSP
MSTRG.30551.1    ------------------------------------------------------------
                                                                             


                        430       440       450       460       470       480
                 =========+=========+=========+=========+=========+=========+
Ec-10_002060     SRKTTSGAPTGAQRAATAAGGIDSEEALEAALLTLPPEERAKSRWTLTVSGDAAVTTAAA
MSTRG.30551.1    ------------------------------------------------------------
                                                                             


                        490       500       510       520       530       540
                 =========+=========+=========+=========+=========+=========+
Ec-10_002060     AAVVATAAAGGPGGTGGRGGGDEARVDLDGAVVVLAGDGGGGSASDGGEGLEALHHHGDD
MSTRG.30551.1    --------------------------------------GGG--G-EEGRGRVALHHHGDD
                                                                #############


                        550       560       570       580       590       600
                 =========+=========+=========+=========+=========+=========+
Ec-10_002060     PDKAGYTPSELVRLARSTVSGQRQLALRALAGVLRFRAACIARGQTPPHRRLPEMLPVAI
MSTRG.30551.1    PGEAGYTPSELVRLARSTVSGQRQLALRALAGVLRIRAACIARGQAPPHPRLPETLSVVL
                 ############################################################


                        610       620       630       640       650       660
                 =========+=========+=========+=========+=========+=========+
Ec-10_002060     RLCLDDGNPSVTSAALNATEALLAPLARDGEDEERAMQGLSWVDYQAFPCEPRPLCTPEE
MSTRG.30551.1    RMCLDDGNPSVASAALSALEAFLAPLARDIEEEENAMQGLSWVDYQALPSEARPLCTPEE
                 ############################################################


                        670       680       690       700       710       720
                 =========+=========+=========+=========+=========+=========+
Ec-10_002060     KQHLPPLKDSAIEEEGDETPGDGGEGEGGKTVRELARLCYEDPCRGLLEMALLPKIASVI
MSTRG.30551.1    KNGLPALRDDGAGEGDEGGDGRDGEETGGKTVGELARLCYEDPCRGMLEMALLPKITSMI
                 ############################################################


                        730       740       750       760       770       780
                 =========+=========+=========+=========+=========+=========+
Ec-10_002060     KS-YADQVAAD----GA-QAASDGGGGLGD--KGSFHGAARATAAATAVSFRPRAALSCL
MSTRG.30551.1    TTNHAGKVAVSTAAGGTHPPASDDAGRSSDLVAGASTAVGRHRGRGADPVIRPRAALSCL
                                     ##########                     #########


                        790       800       810       820       830       840
                 =========+=========+=========+=========+=========+=========+
Ec-10_002060     RVLCAVAGRAPWAAERVASEPGLLDAVREAFLEPRADGTLPLDLSETAPATVTATAIPKQ
MSTRG.30551.1    RVLCAVAGRAPWAAERVAREPGLLDAVREAFLEPSSDGTLPFELSGARSAATSASEQHQP
                 ######################################################      


                        850       860       870       880       890       900
                 =========+=========+=========+=========+=========+=========+
Ec-10_002060     LS---PPPPQEPATSARAEGKARTEGPRDANG---CPPPPPQEQLALLAVRLARVLVQSG
MSTRG.30551.1    PPPRPSSPPQSPTTTSRAPSSAVRGGAHGRRGDGESSAPFEERSAALLAVRLARMLVQSE
                        #########################      ######################


                        910       920       930       940       950       960
                 =========+=========+=========+=========+=========+=========+
Ec-10_002060     RGVALALSQTPLLGSTKGWLALGQGSRRPLPALEAVQREVLELWRCCLSYGVDVAAADTV
MSTRG.30551.1    RGVALLVSRTPLLESARGFLALGQGSKQPLPRLEAVQREVLELWRCCLSYGIDVAAADTV
                 ############################################################


                        970       980       990      1000      1010      1020
                 =========+=========+=========+=========+=========+=========+
Ec-10_002060     VLVARGQGWWPGLSGGVGAAAAAAAAAAPLVADSVTVERGGGGGGGSGGGVGEEPTAAAP
MSTRG.30551.1    VLVARGQGWWPGLDS--------VAARPVLVAD------------------GESAVAAAA
                 #############                                               


                       1030      1040      1050      1060      1070      1080
                 =========+=========+=========+=========+=========+=========+
Ec-10_002060     VGAESVSADSGVGVGDGVGGVGAQSSSGVAQSTSGGAGGGSRGGDSLPPRLAFYRALHQL
MSTRG.30551.1    --VEPAPGDVGVS----VG---------------------GRGG-SLSPKLAFVRALHQL
                                                              ###############


                       1090      1100      1110      1120      1130      1140
                 =========+=========+=========+=========+=========+=========+
Ec-10_002060     ACACVLPPGVSAAG---R-NKNDGAETAGGDGKAGAGAAGTG-VRKEAEGFSVGGVAVVP
MSTRG.30551.1    ACACVLLPGRSSGGSAGSIAGSDGTKPAEGRSDRARGGPGKGPPAAAATTLSLGGLAVVP
                 ##############        ####################     #############


                       1150      1160      1170      1180      1190      1200
                 =========+=========+=========+=========+=========+=========+
Ec-10_002060     ESWADVGDTVDAAVEGALLWLLSGVRGGGRVGGGGAAETAQQAAAVHLVACWVERYQAMS
MSTRG.30551.1    ESTADMGDTVDAAVEATLSWLLG-------------------------------------
                 ######################                                      


                       1210      1220      1230      1240      1250      1260
                 =========+=========+=========+=========+=========+=========+
Ec-10_002060     RTIGRRGSRHLESTAEDAAVLFSSLLASPALPLPAAVAATTAVTRRGDQSGGSGGSGGSG
MSTRG.30551.1    ------------------------------------------------------------
                                                                             


                       1270      1280      1290      1300      1310      1320
                 =========+=========+=========+=========+=========+=========+
Ec-10_002060     SGDGFSQRGVSSGEAAAAAGLDWLSGVFRLGLACERAVPGEAAAALASAPEATAAALAAF
MSTRG.30551.1    ------------------------------------------------------------
                                                                             


                       1330      1340      1350      1360      1370      1380
                 =========+=========+=========+=========+=========+=========+
Ec-10_002060     LAAAGTAGAGAAAADRASAAPTAAVAPPPATFLRRVRRAEAGARWLGCRLLALGCRGGGR
MSTRG.30551.1    ------------------------------------------------------------
                                                                             


                       1390      1400      1410      1420      1430      1440
                 =========+=========+=========+=========+=========+=========+
Ec-10_002060     EGGWPWAALRRDCALQAMALCERGDEAIAVGLLAEALPGSTVDSPEEEGSERPSDDPAAA
MSTRG.30551.1    ------------------------------------------------------------
                                                                             


                       1450      1460      1470      1480      1490      1500
                 =========+=========+=========+=========+=========+=========+
Ec-10_002060     AVGTAAASTSLLRMLFRGAISDPVALSQSSFHLDGRWASLQSLKACTPPGTASLLPLPPH
MSTRG.30551.1    ------------------------------------------------------------
                                                                             


                       1510      1520      1530      1540      1550      1560
                 =========+=========+=========+=========+=========+=========+
Ec-10_002060     WLFLPLASQGGEKAASTAVSACLSLLVQLEREESAYVWSGGGGGGTGCVSPEVKLYHLAN
MSTRG.30551.1    ------------------------------------------------------------
                                                                             


                       1570      1580      1590      1600      1610      1620
                 =========+=========+=========+=========+=========+=========+
Ec-10_002060     ACLYGASVLSGLGVVQHFDWLFSRYLEQCGAGFEGRLAEVIARLALVSAPDPAKMIADAA
MSTRG.30551.1    ------------------------------------------------------------
                                                                             


                       1630      1640      1650      1660      1670      1680
                 =========+=========+=========+=========+=========+=========+
Ec-10_002060     TAAAAAGGRDNNNNGSNSSSSSSARDRARAARRQSHEERALLEFVKDLVASFLADSFGLP
MSTRG.30551.1    ------------------------------------------------------------
                                                                             


                       1690      1700      1710      1720      1730      1740
                 =========+=========+=========+=========+=========+=========+
Ec-10_002060     SFARVLRVFLRTGFPAAARRVVWKELGDVGLLHLLDPPPAGATATAPATDVSSPGSVPAA
MSTRG.30551.1    ------------------------------------------------------------
                                                                             


                       1750      1760      1770      1780      1790      1800
                 =========+=========+=========+=========+=========+=========+
Ec-10_002060     RAINGGGCVADQAYLYPPDVDGAIVEAYLAALEHPRFGPAAAVVVAAAAAAAAAAAATGG
MSTRG.30551.1    ------------------------------------------------------------
                                                                             


                       1810      1820      1830      1840      1850      1860
                 =========+=========+=========+=========+=========+=========+
Ec-10_002060     ERRRHGREEETEGGSALSAGAAAARGLAVEAGRDDSSPSAAAAGGGAVTMREIAVHHFAC
MSTRG.30551.1    ------------------------------------------------------------
                                                                             


                       1870      1880      1890      1900      1910      1920
                 =========+=========+=========+=========+=========+=========+
Ec-10_002060     YLFPRSPHPPPAGGGGGGGGGGGATAPAAPCPPECCKDNQGEPPWRPDFARRKMFERLLR
MSTRG.30551.1    ------------------------------------------------------------
                                                                             


                       1930      1940      1950      1960      1970      1980
                 =========+=========+=========+=========+=========+=========+
Ec-10_002060     QHRESLGRGTGEAGPEVAGSVAAAVLGHHGLGDDSPAAVAADGNKATGERPAIHGEGGAR
MSTRG.30551.1    ------------------------------------------------------------
                                                                             


                       1990      2000      2010      2020      2030      2040
                 =========+=========+=========+=========+=========+=========+
Ec-10_002060     CREGRAWVSELGERRRTLLQAYCRARSGDGRRAVTVAGAGGGDGDGGGGGGGGGGGGVDV
MSTRG.30551.1    --------------------------------------AG--------------------
                                                                             


                       2050      2060
                 =========+=========+===
Ec-10_002060     VSWLEEGRHVEDAVERLAREFFP
MSTRG.30551.1    -----------------------
```

```
Parameters used
Minimum Number Of Sequences For A Conserved Position: 2
Minimum Number Of Sequences For A Flanking Position: 2
Maximum Number Of Contiguous Nonconserved Positions: 8
Minimum Length Of A Block: 10
Allowed Gap Positions: None
Use Similarity Matrices: Yes
```

```
Flank positions of the 8 selected block(s)
Flanks: [528  720]  [741  750]  [772  834]  [848  872]  [879  973]  [1066  1094]  [1103  1122]  [1128  1162]  

New number of positions in Ec-10_002060.aa.fasta.trans.aa_ali.fasta-gb:  470  (22% of the original 2063 positions)
```
